# Anaphylaxis to duck and goose eggs with preserved tolerance to hen’s eggs: A case report 

**DOI:** 10.5414/ALX400691

**Published:** 2026-08-18

**Authors:** Alice Botta, Lea Caron, Matteo Cavara, Alessandra Chiei Gallo, Eleonora Bono, Christian P. Ratti, Sara Pasqualetti, Enrico Iemoli, Valeria Ortolani

**Affiliations:** 1 Allergy and Clinical Immunology Unit, L. Sacco Hospital,; 2 Clinical Pathology Unit, Luigi Sacco University Hospital, ASST Fatebenefratelli-Sacco, and; 3 Department of Biomedical and Clinical Sciences “Luigi Sacco”, Università degli Studi di Milano, Milan, Italy

**Keywords:** anseriformes egg allergy, food allergy, anaphylaxis, hen’s egg tolerance

## Abstract

Background: Selective allergy to eggs from specific avian species, while maintaining tolerance to hen’s eggs, is rare and may present diagnostic challenges. Case report: We report the case of a 75-year-old woman with no history of atopic disease who experienced episodes of anaphylaxis after consuming duck and goose eggs, while tolerating hen’s eggs. Skin prick tests with commercial hen’s egg extracts were negative. Serum testing revealed very low total IgE levels (5 kU/L) and low specific IgE to ovomucoid (Gal d 1), with negative results for other egg proteins. Prick-to-prick testing showed positive reactions to raw duck and goose egg white, whereas results were negative for cooked duck and goose eggs and for both raw and cooked hen’s and quail eggs. An oral food challenge confirmed tolerance to hen’s eggs. Conclusion: This case highlights a rare presentation of selective allergy to eggs from the Anseriformes order with preserved tolerance to hen’s eggs. Comprehensive allergy evaluation – including skin testing, specific IgE measurement, and oral food challenges – may be essential to identify selective sensitizations and to provide appropriate dietary recommendations.

## Introduction 

**Figure 1.**
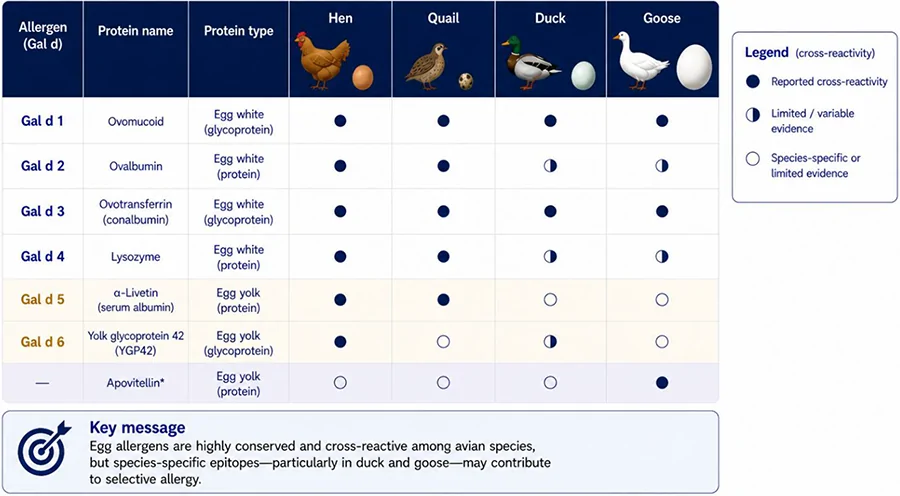
Main egg allergens and their potential cross-reactivity across selected avian species.

Allergy to hen’s eggs (*Gallus gallus domesticus*) is one of the most common food allergies, particularly in early childhood. The proteins that trigger allergic responses are found in both egg white and yolk. Specifically, in the egg white, the main allergens are ovomucoid (Gal d 1), ovalbumin (Gal d 2), ovotransferrin (Gal d 3), lysozyme (Gal d 4), while in the yolk, they are α-livetin (serum albumin, Gal d 5) and yolk glycoprotein 42 (vitellogenin, Gal d 6) [1]. Cross-reactivity has been reported among egg proteins from different avian species, particularly egg white proteins (Figure 1) [2]. Individuals allergic to hen’s eggs are most commonly sensitized to other species including quail, duck, turkey, partridge, pigeon, and goose. This appears to be associated with the shared evolutionary origin of these species [3], necessitating dietary elimination not only for hen’s eggs but also for eggs from other species. However, a few cases of individuals experiencing IgE-mediated allergic reactions after ingesting eggs from various avian species, without exhibiting an allergy to hen’s eggs, have been described in the literature. The responsible protein appeared to be ovalbumin, with specific antigenic determinants for the Anseriformes order, which are not present in the ovalbumin of the Galliformes order [4]. A duck ovalbumin was also identified as a potential responsible allergen, likely recognized by IgE due to specific epitopes not present in hen ovalbumin [5]. In a different case, a lysozyme with specific antigenic determinants for the Anseriformes order has been identified as a potential responsible allergen [6]. Recently, the serum of a patient who exhibited an allergic reaction to goose eggs but not to hen’s eggs was studied. A possible responsible allergen was identified as apovitellin, present in the yolk and likely influenced in its allergenicity by the cooking temperature of the food [7]. Other researchers have identified a protein present in the yolk, vitellogenin, as a possible cause of selective anaphylaxis to duck egg [8]. 

## Case report 

In June 2025, a 75-year-old woman with no history of atopic disease was referred to us after experiencing a reaction ~ 7 months earlier, triggered by the ingestion of hard-boiled goose eggs. The reaction was characterized by general malaise, dyspnea, speech impairment, profuse sweating, multiple episodes of vomiting, and diarrhea, occurring about an hour after consuming the eggs. Approximately 15 days after the initial episode, she consumed goose eggs again, experiencing a recurrence of similar symptoms. On both occasions, she did not seek emergency care, and the symptoms resolved spontaneously. In June 2025, approximately an hour after consuming a slice of homemade apple pie, the woman experienced profuse sweating, nausea, vomiting, and diarrhea. She sought emergency care, where she was administered intravenous fluids. Subsequently, the woman discovered that the apple pie contained either duck or possibly goose egg. She was able to tolerate apples but avoided eggs and egg-containing products. She also tolerated chicken meat. At the time of the reactions, the patient was receiving treatment with alirocumab, clopidogrel, pregabalin, and melatonin, with no recent changes in therapy. No relevant cofactors were reported in temporal association with the reactions. She reported having previously consumed duck and goose eggs on rare occasions, but not as part of her regular diet, and denied any relevant environmental exposure to ducks, geese, or related products (including feathers, pillows, or bedding). 

The woman initially underwent skin prick tests (SPTs) using commercial extracts of hen’s egg yolk and egg white (Lofarma, Milan, Italy), which produced negative results. Subsequent blood tests revealed total IgE levels of 5 kU/L. Specific IgE levels were measured using the ImmunoCAP system (Thermo Fisher Scientific, Uppsala, Sweden), showing egg white 0.08 kU/L and ovomucoid (Gal d 1) 0.38 kU/L, with negative results for egg yolk, ovalbumin (Gal d 2), ovotransferrin (Gal d 3), and lysozyme (Gal d 4). Baseline serum tryptase was within normal limits; however, acute-phase tryptase was not measured during the acute reaction in the emergency department. 

**Figure 2.**
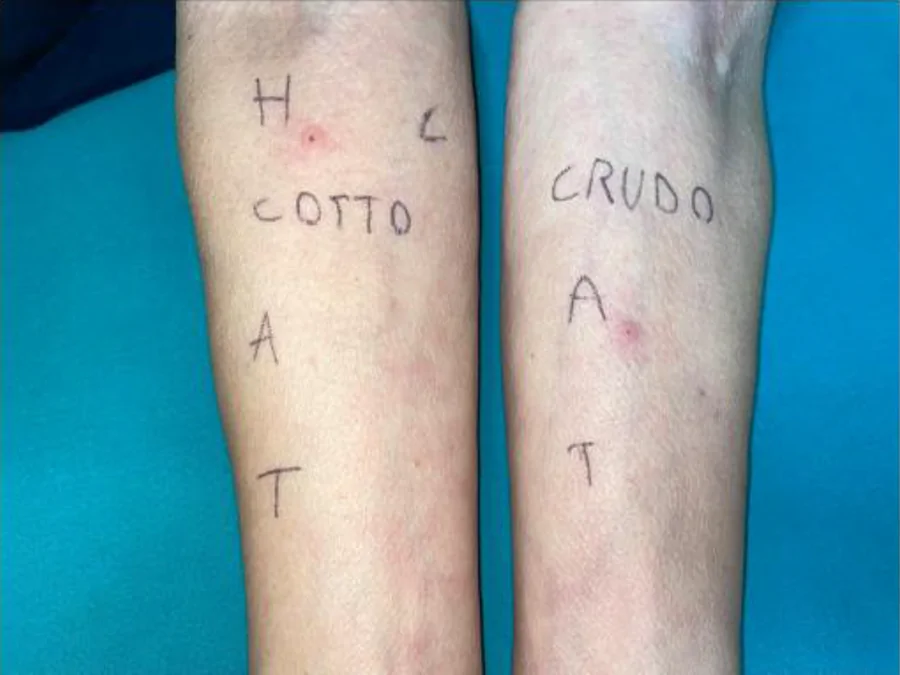
Prick-by-prick test with goose egg. Testing was carried out with cooked (“cotto”) and raw (“crudo”) egg. H: histamine (positive control); C: negative control; A: egg white (albumen); T: egg yolk. A positive wheal (6 mm) is observed in response to raw goose egg white.

A prick-to-prick test with fresh cooked and raw eggs was performed, using eggs of duck (*Anas domesticus*), goose (*Anser anser domesticus*), hen (*Gallus gallus domesticus*), and quail (*Coturnix coturnix*). Prick-to-prick testing was positive to raw duck egg white (average wheal size of 8 mm) and raw goose egg white (average wheal size of 6 mm), while it was negative for cooked duck egg and goose egg white and yolk, as well as for raw and cooked hen and quail egg white and yolk (Figure 2). 

Since the prick-to-prick test yielded negative results for hen’s egg, the patient underwent an oral food challenge with cooked (hard-boiled) and raw hen’s egg yolk and egg white, which she tolerated at the cumulative dose equivalent to 1 whole hen’s egg (approximately 60 g, corresponding to about 7.4 g of protein). The patient declined an oral food challenge with duck or goose egg given the severity of her symptoms and the positive prick-to-prick test results to raw egg (despite negative results with cooked egg). She was discharged with instructions to avoid duck and goose eggs but could consume both raw and cooked hen’s eggs. 

Our affiliated university hospital laboratory attempted SDS-PAGE and immunoblotting, but the analysis was unsuccessful, possibly due to the very low total IgE level (5 kU/L) and consequently low allergen-specific IgE levels [9]. 

## Discussion 

This case report highlights a rare instance of anaphylaxis triggered by the consumption of duck and goose eggs while maintaining tolerance to hen’s eggs. The woman’s symptoms, which included general malaise, dyspnea, speech difficulty, profuse sweating, vomiting, and diarrhea, occurred shortly after consuming the offending eggs and a homemade apple pie that contained these eggs. 

The diagnostic process involved various tests to identify the specific allergens responsible for her reactions. SPTs using commercial extracts of hen’s egg yolk and egg white yielded negative results. Subsequent blood tests revealed specific IgE levels for ovomucoid (Gal d 1), a common egg white protein. 

A prick-to-prick test was conducted with various fresh cooked and uncooked eggs, including those from duck, goose, hen, and quail. The test showed a positive reaction only to raw duck egg white and raw goose egg white, indicating a specific sensitivity to proteins found in duck and goose eggs. However, the test was negative for cooked duck and goose eggs and all other tested egg types, including hen and quail. 

To confirm her tolerance to hen’s eggs, an oral food challenge was performed with both cooked and raw hen’s egg yolk and egg white, which she successfully tolerated. 

Cross-reactivity among egg proteins from different avian species has been reported, particularly among the egg white of various species. However, it is possible that some proteins are specific to individual species, allowing an individual who is sensitized to one of these proteins to tolerate eggs from other species. Regarding duck and goose, both belong to the Anseriformes order, and their egg whites are similar [2]. 

From a compositional standpoint, the total protein content varies among avian eggs primarily as a consequence of differences in egg size rather than marked variations in protein concentration. A typical hen’s egg contains approximately 6 – 7 g of protein, whereas duck eggs contain about 8 – 9 g and goose eggs approximately 18 – 20 g per egg, reflecting their larger overall mass. Quail eggs contain approximately 1.0 – 1.4 g of protein per egg [10]. 

In our case, the patient exhibited a reaction to both duck and goose eggs, and tests confirmed sensitization to duck and goose eggs. 

Attempts to identify the responsible allergen by SDS-PAGE and immunoblotting were unsuccessful. This may be explained by the very low total IgE level observed in our patient (5 kU/L), which may have limited the sensitivity of the assay and hindered the detection of specific IgE-binding proteins. 

The specific allergen responsible remains unidentified; however, our patient exhibited a reaction to cooked egg, suggesting the potential involvement of a heat-resistant protein. Considering the prick-to-prick test results (positive only for raw eggs and not for cooked eggs), and taking into account the patient’s reaction after ingesting a cake containing duck eggs, another hypothesis is that the implicated allergen might be heat-sensitive, and the apple pie was not sufficiently cooked to alter the characteristics of the allergen, and that the goose egg had not been boiled sufficiently, as it requires a longer boiling time than a hen’s egg due to its larger size. The discrepancy between in vivo testing and clinical reactivity remains unexplained and is more likely to reflect differences in allergen stability and food processing conditions. In particular, food matrix effects and heterogeneous heat distribution in baked products may contribute to the persistence of allergenic epitopes despite cooking. 

Similar cases reported in the literature have led to the proposal of various hypotheses concerning the implicated proteins, notably implicating egg white proteins (ovalbumin, lysozyme) [4, 5, 6] and yolk proteins (apovitellin, vitellogenin) as potential culprits [7, 8]. Among these allergens, lysozyme (Gal d 4) is recognized for its relative heat stability and may represent a potential candidate in the present case, despite negative serum specific IgE results, possibly reflecting limitations in detection or differences in allergenic epitopes across avian species [11]. 

Further studies would be beneficial to deepen our understanding of the specific proteins involved in allergic reactions to eggs from different avian species and elucidate their characteristics, including heat resistance. 

## Conclusion 

This case highlights a rare instance of selective allergy to eggs from the Anseriformes order in a patient who tolerates hen’s eggs, emphasizing the importance of comprehensive allergy testing, including both skin and blood tests, as well as oral food challenges when appropriate. This approach allows clinicians to identify selective sensitizations and guide safe dietary recommendations. 

## Acknowledgment 

We sincerely thank our patient for her consent and willingness to publish her case. 

## Consent for publication 

Written informed consent for publication of this case report was obtained from the patient. 

## Authors’ contributions 

AB, MC, and LC contributed equally to the conception and design of the study and to the drafting of the manuscript. CPR, EB, and ACG contributed to the literature search and data collection. SP contributed to the laboratory analyses. EI and VO revised the manuscript for intellectual content. All authors read and approved the final version of the manuscript. 

## Funding 

No funding was received for this study. 

## Conflict of interest 

The authors declare no conflict of interest.
